# Dioscin Reduces Vascular Damage in the Retina of *db/db* Mice by Inhibiting the VEGFA Signaling Pathway

**DOI:** 10.3389/fphar.2021.811897

**Published:** 2022-01-28

**Authors:** Jun Wang, Guang Yan Yang, Hong Yan Sun, Ting Meng, Chu Chu Cheng, Hui Pan Zhao, Xiao Ling Luo, Ming Ming Yang

**Affiliations:** ^1^ Department of Endocrinology, Shenzhen People’s Hospital, The Second Clinical Medical College of Jinan University, The First Affiliated Hospital of Southern University of Science and Technology, Shenzhen, China; ^2^ Department of Ophthalmology, Shenzhen People’s Hospital, The Second Clinical Medical College of Jinan University, The First Affiliated Hospital of Southern University of Science and Technology, Shenzhen, China

**Keywords:** diabetic retinopathy, angiogenesis, retinal microvascular endothelial cells, VEGFA, dioscin

## Abstract

Diabetic retinopathy (DR) is a complication of diabetes that has a serious impact on the quality of life of patients. VEGFA is necessary in the physiological state to maintain endothelial activity and physical properties of blood vessels. VEGFA plays an important role in the promotion of neovascularization; therefore, inhibition of VEGFA can degrade the structure of blood vessels and reduce neovascularization. In the present study, HERB, a high-throughput experimental and reference-oriented database of herbal medicines, was used for compound mining targeting VEGFA. The compounds most likely to interact with VEGFA were screened by molecular docking. Next, the compounds were used to verify whether it could inhibit the activity of the VEGF signaling pathway *in vitro* and neovascularization *in vivo*. *In vitro*, we found that dioscin could inhibit the activation of the VEGFA–VEGFR2 signaling pathway and cell proliferation of human retinal microvascular endothelial cells in a high-glucose (HG) environment. A more important dioscin intervention inhibits the expression of pro-angiogenic factors in the retinas of *db/db* mice. In conclusion, our study indicates that dioscin reduces the vascular damage and the expression of pro-angiogenic factors in the retina of *db/db* mice and implies an important and potential application of dioscin for treatment of DR in clinics.

## Introduction

Diabetes mellitus is a rapidly growing global disease that affects approximately 415 million people and is expected to reach 642 million by 2040 (Saeedi et al., 2019). Diabetic retinopathy (DR) is a complication of diabetes that has a serious impact on the quality of life of patients (Cheung et al., 2010).

VEGF is necessary in the physiological state to maintain endothelial activity and physical properties of blood vessels (Senger, 2010). Under pathological conditions, ischemia and hypoxia cause excessive production of VEGF by retinal epithelial cells, endothelial cells, and the retinal pigment epithelium, while VEGF recruits leukocyte adhesion to aggravate ischemia and hypoxia, creating a vicious circle (Virgili et al., 2017). Overexpression of VEGF leads to proliferation of the retinal pigment epithelium, proliferation of vascular endothelial cells, and increased permeability; this promotes the activation of inflammatory factors, leading to retinal hemorrhage, exudation, and macular edema, and can even lead to the formation of neovascularization (Osaadon et al., 2014). VEGF plays an important role in the formation of neovascularization; therefore, inhibition of VEGF can degrade the structure of blood vessels and reduce neovascularization (Ajlan et al., 2016).

Anti-VEGF drugs currently used in clinical practice mainly inhibit neovascularization by inhibiting the binding of VEGF to its receptor. Macugen specifically binds to VEGF165 and inhibits the binding of VEGF to its receptor to reduce vascular permeability and neovascularization (Huang et al., 2016). Bevacizumab can improve vision in patients with DR by combining with VEGFA (Wells et al., 2016). Conbercept inhibits endothelial cell proliferation and neovascularization by binding to VEGFA and VEGFB receptors (Xia et al., 2021). Despite this, there are also concerns about the limitations and adverse effects of anti-VEGF injection therapy. Monthly or bi-monthly injections are required to ensure efficacy due to the short half-life of anti-VEGF drugs. Endophthalmitis is a rare adverse effect of vitreous cavity injections, the incidence of which can be increased by frequent injections (Wang and Lo, 2018). Therefore, it is important to develop an easy-to-use anti-VEGF therapy with few adverse effects.

In mammals, the VEGF family consists of five secreted proteins, VEGF-A, -B, -C, -D, and placental growth factor, of which the role of VEGF-A in the endothelium has been most intensively studied (Matsumoto and Ema, 2014). VEGF-A interacts with vascular endothelial growth factor receptor 1 and 2 (VEGFR1 and VEGFR2), which regulate most aspects of the endothelial response, such as the proliferation and migration of ECs and the permeability of blood vessels (Blanco and Gerhardt, 2013). VEGFA has been shown to promote angiogenesis in healing wounds by regulating FOXO1 (Jeon et al., 2018). MicroRNA-140-5p inhibits breast cancer invasion and angiogenesis by targeting VEGF-A (Lu et al., 2017). B7-H3 induces VEGFA expression through activation of the NF-κB pathway and promotes colorectal cancer angiogenesis (Wang et al., 2020). The abovementioned studies illustrate that VEGFA is closely associated with neovascularization; therefore, anti-VEGFA may be an effective treatment for DR.

In the present study, HERB (http://herb.ac.cn/) (Fang et al., 2021), a high-throughput experimental and reference-oriented database of herbal medicines, was used for compound mining targeting VEGFA. The compounds most likely to interact with VEGFA were screened by molecular docking. Next, the compounds were used to verify whether it could inhibit the activity of the VEGF signaling pathway *in vitro* and neovascularization *in vivo*.

## Materials and Methods

### *In vivo* Studies With Animals

All animal care and experimental protocols for *in vivo* studies conformed to the Guide for the Care and Use of Laboratory Animals published by the NIH (NIH publication no. 85-23, revised 1996). We decided the sample size for animal studies based on a survey of data from published research or preliminary studies, and no mice were excluded from the statistical analysis. Animal studies were approved by the Ethics Committee of the Second Clinical Medical College of Jinan University, Shenzhen People’s Hospital (IACUC Issue No: 20200319-45). Male type 2 diabetic (BKS.C g-m +/+ Lepr^*db*^/j, *db/db*) mice and C57BLKS/J wild-type mice at the age of 6 weeks were purchased from Gempharmatech Co. Ltd. (Nanjing, Jiangsu, China). They were maintained in SPF units of the Animal Center of Shenzhen People’s Hospital (with a 12-h light cycle from 8 a.m. to 8 p.m., 23 ± 1°C, 60–70% humidity) and maintained on a standard rodent diet with free access to water in plastic bottles. The mice were allowed to acclimatize to their housing environment for at least 7 days before the experiments. Up to five mice were housed in each plastic cage with corncob bedding material. We conducted the treatment in a blinded fashion. The drugs used for treating animals were prepared by researchers who did not undergo treatment. The animals were randomized before receiving treatment. Dioscin was delivered by oral and ocular administration. Doses of oral administration [80 mg/kg body weight (mpk)] (Wu et al., 2021) and ocular delivery (0.32 μg/kg) were applied; Treatment lasted for 16 weeks (from 8-week-old to 24-week-old *db/db* mice). The mice in the normal group received vehicle as the control. At the end of the experiment, all mice were anesthetized and euthanized in a CO_2_ chamber, followed by collection of eyeball samples.

### Cell Culture

Human retinal microvascular endothelial cells (HRMECs) were obtained from the American Type Culture Collection (ATCC, Manassas, VA, United States). The cells were cultured in DMEM+ 10% fetal bovine serum (FBS) (Gibco, NY, United States) at 37 °C in a 5% CO_2_ atmosphere. 5  mM (normal glucose) or 22 mM (high glucose) of D-glucose (Gibco) were added to the stimulation medium for 48  h.

### Molecular Docking

Discovery Studio (DS) was used for molecular docking of VEGFA and its compounds. DS 2019 version is molecular modeling software for protein structure studies and drug discovery (Zhang et al., 2020). The structures of small-molecule compounds and VEGFA were downloaded from the Protein Data Bank (PDB) database (https://www.rcsb.org) (Karuppasamy et al., 2020). First, the compounds were used for ligand preparation, a method to remove duplicates, enumerate isomers and tautomers, and generate 3D conformations. Next, a series of preparations were also applied to the protein receptor, including removing water molecules, adding hydrogen atoms, and setting up active pockets. Finally, CDocker was used for molecular docking, an algorithm that allows precise docking of any number of ligands to a single protein receptor (Wu et al., 2003). -CDOCKER interaction energy (CIE) reflects the ability of ligands and receptors to interact in molecular docking.

### Determination of Fasting Blood Glucose Levels

During the treatment, blood was withdrawn from the mouse tail vein after overnight fasting at different time points. Blood glucose levels were determined using a OneTouch glucometer and test strips (LifeScan) according to the manufacturer’s instructions.

### Quantitative Real-Time Polymerase Chain Reaction

At the end of the experiment, TRIzol reagent (Invitrogen) was used to extract total RNA as described in the classic protocol. Chloroform was mixed well with the homogenate and centrifuged at 13,300 rpm for 15 min at 4°C. The top aqueous phase was collected, mixed with isopropanol, and stored at −20°C. The next day, RNA was centrifuged and washed with 75% ethanol and 100% ethanol in sequence. RNA was dissolved in an appropriate amount of RNase water. cDNA was obtained using a reverse transcription kit purchased from New England Biolabs (Ipswich, MA, United States). qPCR was performed using the ABI StepOnePlus^TM^ Real-Time PCR system (Applied Biosystems) with specific primers (Table 1). The relative mRNA levels of target genes were analyzed using Equation, 2^–ΔCt^ (ΔCt = Ct of the target gene—Ct of β-actin) and normalized using the level detected in the control group as 1.

**TABLE 1:** Sequences of primers for qPCR analysis.

Gene	Forward	Backward
hKi67 (ID: 4288)	GCC​TGC​TCG​ACC​CTA​CAG​A	GCT​TGT​CAA​CTG​CGG​TTG​C
hPCNA (ID: 5111)	ACA​CTA​AGG​GCC​GAA​GAT​AAC​G	ACA​GCA​TCT​CCA​ATA​TGG​CTG​A
hHIF1a (ID: 3091)	CAC​CAC​AGG​ACA​GTA​CAG​GAT	CGT​GCT​GAA​TAA​TAC​CAC​TCA​CA
mVegfa (ID: 22339)	CTG​CCG​TCC​GAT​TGA​GAC​C	CCC​CTC​CTT​GTA​CCA​CTG​TC
mAngpt2 (ID: 11601)	CCT​CGA​CTA​CGA​CGA​CTC​AGT	TCT​GCA​CCA​CAT​TCT​GTT​GGA
mHif1a (ID: 15251)	ACC​TTC​ATC​GGA​AAC​TCC​AAA​G	CTG​TTA​GGC​TGG​GAA​AAG​TTA​GG
mVegfr2 (ID: 16542)	TTT​GGC​AAA​TAC​AAC​CCT​TCA​GA	GCA​GAA​GAT​ACT​GTC​ACC​ACC

### Western Blotting

After treatment, a piece of tissue or cell was lysed or homogenized in lysis buffer (Sigma-Aldrich, St. Louis, MO, United States), and total protein was obtained according to the classical protocol. 1:1000 diluted fresh primary antibody or anti–β-actin antibody (Santa Cruz Biotechnology; 1:5000) in PBS containing 1% fresh dry fat-free milk and 1:5000 diluted fresh HRP-conjugated anti-Rabbit or mouse IgG in PBS containing 1% fresh dry fat-free milk were used. Western blotting experiments were performed using antibodies against VEGFA (Abcam), VEGFR2 (Cell Signaling Technology), phosphorylation of ERK1/2 (Cell Signaling Technology), ERK1/2 (Cell Signaling Technology), Akt (Cell Signaling Technology), and Akt (Cell Signaling Technology), as described previously.

### Immunoprecipitation

To determine the interaction of VEGFA and VEGFR2 in HRMEC homogenates, 1 mg whole-cell extracts was incubated with antibodies against VEGFA (Abcam) overnight under stringent conditions, immunopurified using Dynabeads Protein G beads (Life Technologies Corporation), and immunoblotted using VEGFR2 (Cell Signaling Technology).

### Preparation and PAS Staining of the Retina

Vasculature and quantitation of acellular capillaries retinal vasculature were performed according to a previously described method (Bell et al., 2008) with minor modifications. Briefly, mouse eyes were fixed in 4% paraformaldehyde freshly prepared in PBS (PFA/PBS) overnight after enucleation. The retinas were dissected from eyeballs, washed in water overnight with gentle shaking at room temperature (RT), and then digested in 3% trypsin solution (Invitrogen, Grand Island, NY) for 2–3 h at 37°C. The tissue was then transferred into filtered water, and the network of vessels was freed from adherent retinal tissue by gentle shaking and manipulation under a dissection microscope. The vessels were then mounted on clean slides, air-dried completely, and stained with periodic acid Schiff (PAS) solution according to the manufacturer’s instructions. After the tissue was stained and washed in water, it was dehydrated and mounted using Permount Mounting Medium (Fisher Scientific, Pittsburgh, PA). The retinal vessels were observed and photographed under a microscope. The density of PAS staining was quantified using Photoshop software. Acellular capillaries were randomly counted in three field areas around the mid-retina. Acellular capillaries were defined as capillary-sized vessel tubes with no nuclei along their lengths (Feit-Leichman et al., 2005). Data are presented as the number of acellular capillaries per 0.1 mm^2^ of the retina.

### Data Analysis

All experiments were repeated at least thrice, and representative results are presented. All data are presented as mean ± standard error and were analyzed by Student’s-test or one-way ANOVA with Bonferroni correction. The differences were considered significant at *p* < 0.05.

## Results

### Dioscin Inhibits HG-Induced Phosphorylation of VEGFR2 in Human Retinal Microvascular Endothelial Cells

**FIGURE 1:**
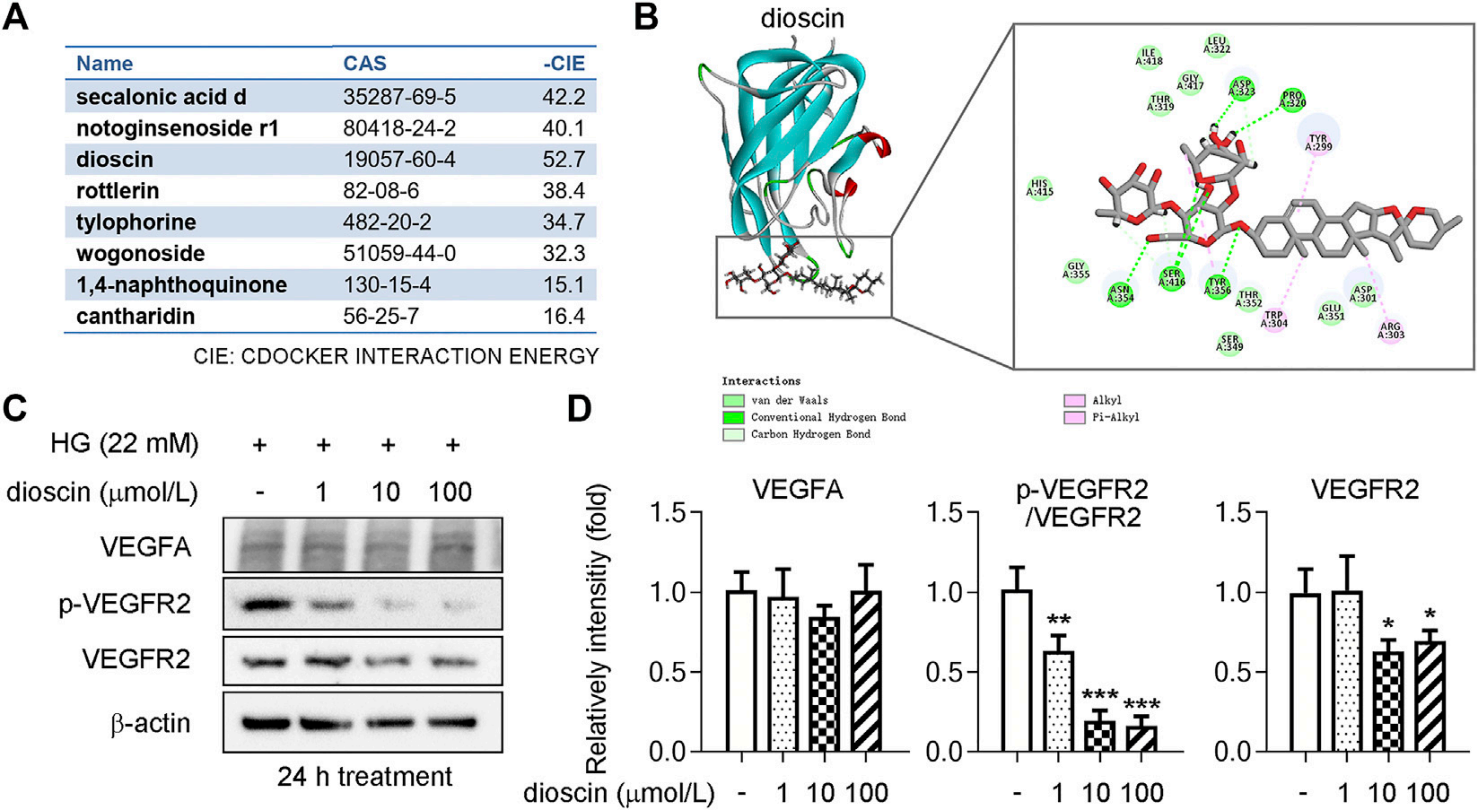
Dioscin inhibits phosphorylation of VEGFR2 in human retinal microvascular endothelial cells. **(A)** Eight compounds were predicted through the HERB website to target VEGFA, including CAS numbers and -CIE. **(B)** Interaction of dioscin with VEGFA as simulated by DS software. The box indicates the 2D structure of the two interactions, the circle indicates the amino acid, and the dashed line indicates the forces that sustain the interaction. **(C, D)** Protein level of VEGFA and phosphorylation of VEGFR2 (p-VEGFR2) and VEGFR2 was detected by Western blot **(C)**. Quantitative results of panel C are shown in panel D, *n* = 3. *p < 0.05, **p < 0.01, and ***p < 0.001 vs. the control group by one-way ANOVA with Bonferroni correction.

HERB (http://herb.ac.cn/), a high-throughput experimental and reference-oriented database of herbal medicines, was used for compound mining targeting VEGFA (Fang et al., 2021). We identified eight compounds with CAS numbers that target VEGFA in this database (Figure 1A). Discovery Studio (DS) 2019 is molecular modeling software for protein structure studies and drug discovery (Zhang et al., 2020). Secalonic acid d, notoginsenoside r1, dioscin, rottlerin, tylophorine, wogonoside, 1,4-naphthoquinone, and cantharidin with VEGFA had -CDOCKER Interaction Energies (CIE) of 42.2, 40.1, 52.7, 38.4, 34.7, 32.3, 15.1, and 16.4 kcal/mol (Figure 1A). According to previous publications on drug–protein interactions, compounds with a -CIE of approximately 50 kcal/mol can be used as effect moles for their putative targets (Li et al., 2013; Yan et al., 2019). Molecular docking analysis revealed that dioscin was the most likely compound to interact with VEGFA and that the main factors sustaining their amino acid interactions were hydrogen bonds, van der Waals forces, and alkyl groups (Figure 1B).

To determine whether dioscin could inhibit HG-induced activation of the VEGFA signaling pathway, human retinal microvascular endothelial cells (HRMECs) were treated with different doses of dioscin (1, 10, and 100 μmol/L) in the presence of high glucose. Our results showed that dioscin treatment did not influence the protein level of VEGFA when compared with HG treatment alone (control group) (Figure 1C). The main VEGF receptor in endothelial cells is VEGFR2. VEGFR2 is essential for endothelial cell biology during development and in adults in physiology and pathology (Ferrara et al., 2003). On VEGFA binding to VEGFR2, signaling molecules bind to phosphorylation sites in the intracellular domain of VEGFR2 and activate downstream mediators, resulting in biological responses such as proliferation, migration, survival, and permeability (Ferrara et al., 2003). When compared with the control group, 1 μmol/L dioscin treatment inhibited the phosphorylation of VEGFR2. Likewise, 10 and 100 μmol/L dioscin treatment significantly inhibited the phosphorylation (activation) of VEGFR2 compared to the control group. Interestingly, there was no significant difference between the control group and the 1-μmol/L dioscin treatment group. However, 10 or 100 μmol/L dioscin treatment significantly inhibited VEGFR2 protein levels when compared with the control group.

### Dioscin Inhibits HRMEC Proliferation in HG Environment

**FIGURE 2:**
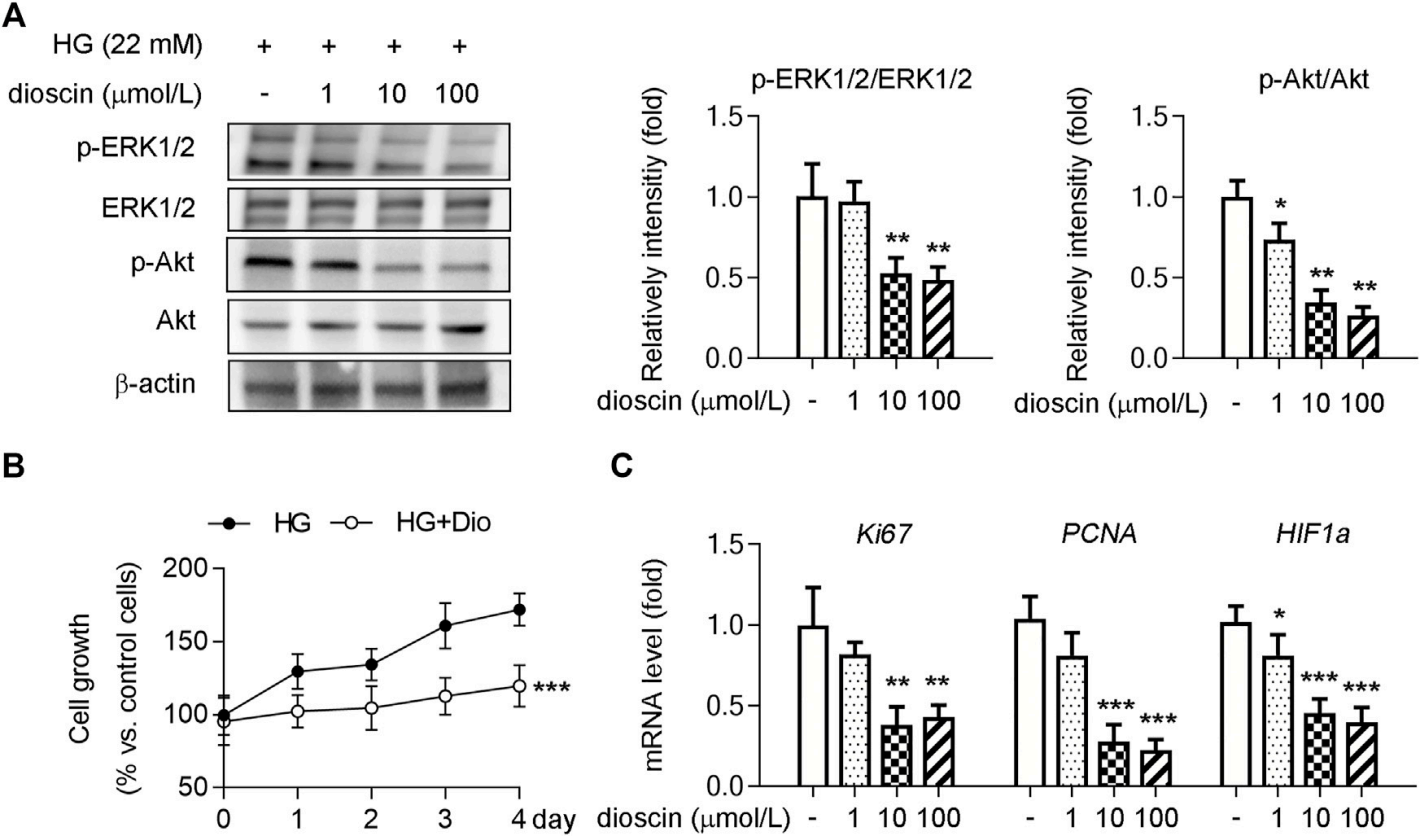
Dioscin inhibits HRMEC proliferation in an HG environment. HRMECs treated with dioscin as indicated in panel A. **(A)** Protein levels of p-ERK1/2, ERK1/2, p-Akt, and Akt were detected by Western blot, and the quantitative results are shown in the right panel, *n* = 3. **(B)** Cell growth of HRMECs was assessed using a CCK-8 assay, n = 3. **(C)** MRNA levels of *Ki67, PCNA*, and *HIF1α* were determined by qPCR, *n* = 5. *p < 0.05, **p < 0.01, and ***p < 0.001 vs. the control group by one-way ANOVA with Bonferroni correction.

Cell proliferation–related Akt and ERK1/2 signaling pathways are the main downstream signaling cascades of the VEGFA–VEGFR2 signaling pathway (Zhang et al., 2019; Wu et al., 2020). To determine whether dioscin could inhibit proliferation in the presence of HG, the phosphorylation of ERK1/2, Akt, and the protein levels of ERK1/2 and Akt were measured. When compared with the control group, 1 μmol/L dioscin significantly decreased the phosphorylation of Akt (Figure 2A). Similarly, treatment with 10 and 100 μmol/L dioscin inhibited the phosphorylation of ERK1/2 and Akt compared to the control group (Figure 2A). In addition, treatment with 10 μmol/L dioscin significantly inhibited cell growth compared to the control group (Figure 2B). The mRNA levels of cell proliferation–related genes (*Ki67 and PCNA*) and *HIF1α* (the main inducer of VEGF) also supported our finding that dioscin treatment could inhibit cell proliferation (Figure 2C). Consistent with these results, dioscin inhibited the proliferation of HRMECs in an HG environment.

### Dioscin Regulates Phosphorylation of VEGFR2 Through Inhibition of VEGFA Interaction With VEGFR2

**FIGURE 3:**
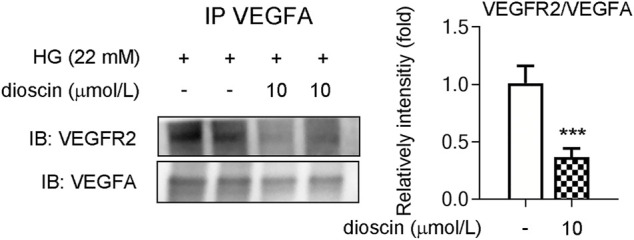
Dioscin inhibits the interaction between VEGFA and VEGFR2. HRMECs treated with dioscin as indicated in panel A. Equal amounts of protein were subjected to immunoprecipitation with VEGFA antibody, followed by immunoblotting with antibody against VEGFR2. The quantitative results are shown in the right panel, *n* = 3. ***p < 0.001 vs. the control group by Student’s *t*-test.

**FIGURE 4:**
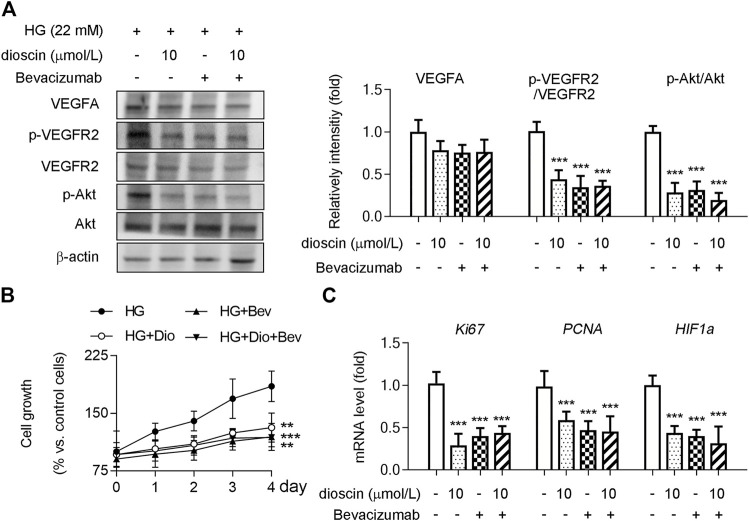
Dioscin regulates the VEGFA–VEGFR2 signaling pathway. HRMECs treated with dioscin and/or bevacizumab as indicated in panel A. **(A)** Protein level of VEGFA, p-VEGFR2, VEGFR2, p-Akt, and Akt was detected by Western blot, and the quantitative results were shown in the right panel, *n* = 3. **(B)** Cell growth of HRMECs was assessed using a CCK-8 assay, *n* = 3. **(C)** MRNA levels of *Ki67, PCNA*, and *HIF1α* were determined by qPCR, *n* = 5. ***p < 0.001 vs. the control group by one-way ANOVA with Bonferroni correction.

Although it was observed that dioscin could inhibit the activation of the VEGFA signaling pathway, no effect of dioscin on the protein level of VEGFA was observed. Hence, we hypothesized that dioscin might influence the binding of VEGFA and VEGFR2. Co-immunoprecipitation showed that dioscin treatment inhibited VEGFA interaction with VEGFR2 (Figure 3). To further determine whether dioscin regulates the interaction between VEGFA and VEGFR2, bevacizumab and/or dioscin were used to treat HRMECs (Figure 4A). Bevacizumab, a humanized monoclonal antibody, specifically binds to all VEGFA isoforms with high affinity and inhibits their interaction with VEGFR1 and VEGFR2 (Tan et al., 2017). Our results showed that bevacizumab or dioscin treatment significantly decreased the phosphorylation levels of VEGFR2 and Akt when compared with the control group (Figure 4A). Interestingly, there was no significant difference in the phosphorylation of VEGFR2 and Akt between bevacizumab treatment and bevacizumab + dioscin treatment in the HG environment (Figure 4A). Consistent with this, similar results were observed; bevacizumab or dioscin treatment inhibited cell growth compared with the control group (Figure 4B). In addition, bevacizumab and dioscin co-treatment did not further decrease cell growth when compared with bevacizumab or dioscin treatment (Figure 4B). Finally, the mRNA levels of *Ki67, PCNA*, and *HIF1α* were determined, and similar results were observed (Figure 4C).

### Dioscin Inhibit Vascular Damage in the Retina of *db/db* Mice

**FIGURE 5:**
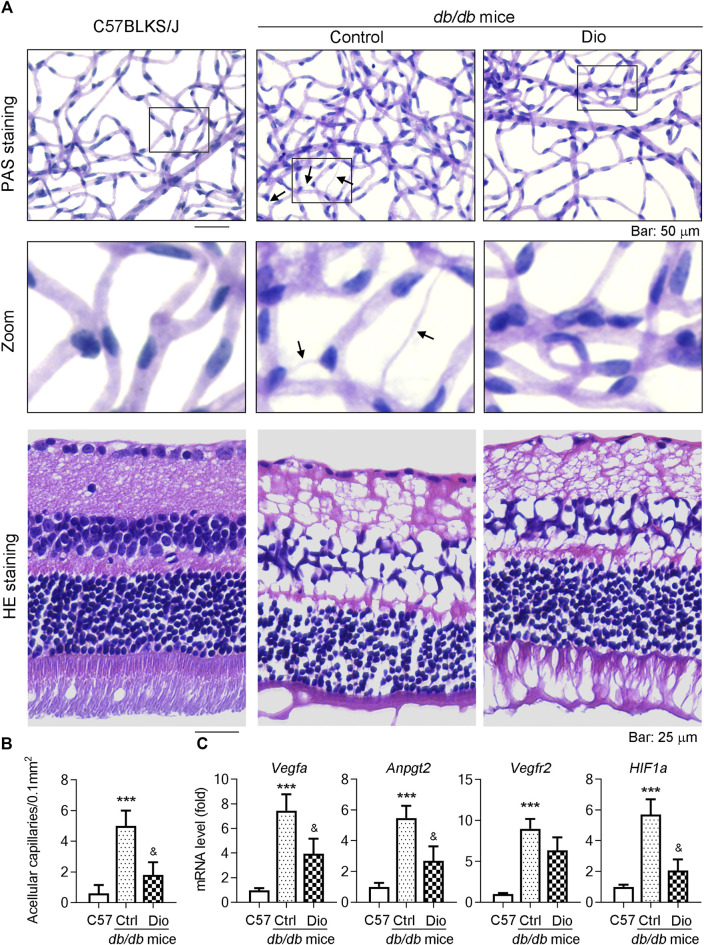
Ocular delivery of dioscin ameliorates vascular damage in the retina of db/db mice. **(A)** At the end of the study, mouse eyes were collected and the retinal vascular network was prepared, followed by PAS and H and E staining. The representative images from each group are presented. Black arrows indicate acellular capillaries in the retinal vasculature. Bars: 50 μm. **(B)** Quantitation of acellular capillaries in the retina. **(C)** *Vegfa*, *Vegfr2, Angpt2,* and *Hif1α* mRNA expression in the retinas was determined by qPCR analysis, *n* = 5. ***p < 0.001 vs. C57BLKS/J mice and ^&^ *p <* 0.05 vs. *db/db* mice by one-way ANOVA with Bonferroni correction.

**FIGURE 6:**
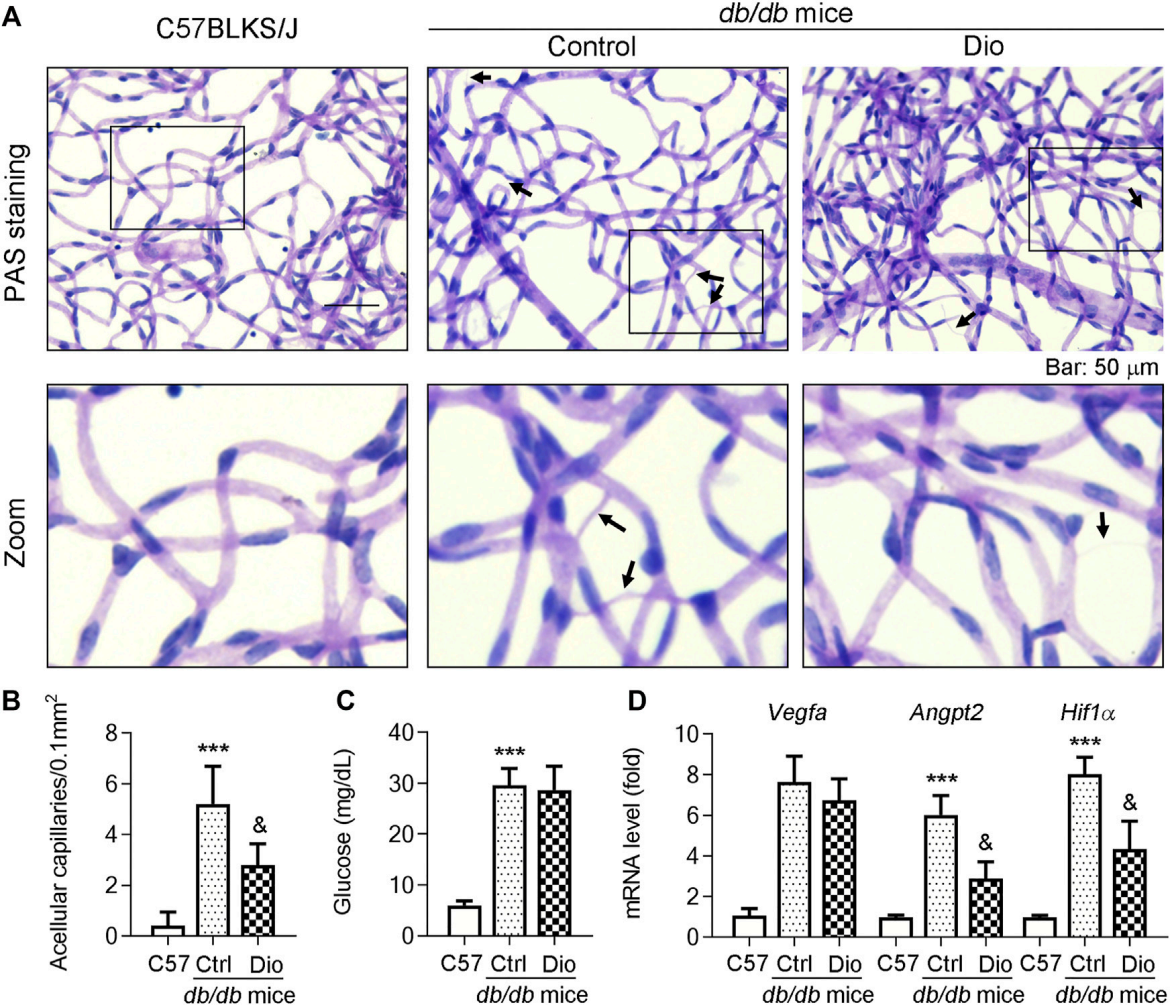
Oral administration of dioscin inhibits vascular damage in the retina of *db/db* mice. The *db/db* mice were administrated with 80 mpk of dioscin daily from 8 weeks of age to 24 weeks of age. **(A)** At the end of the study, mouse eyes were collected and the retinal vascular network was prepared, followed by PAS staining. The representative images from each group are presented. Black arrows indicate acellular capillaries in the retinal vasculature. Bars: 50 μm. **(B)** Quantitation of acellular capillaries in the retina. **(C)** At the end of study, mice were starved for 16 h and blood samples were collected and the glucose levels were determined, *n* = 5. **(D)** *Vegfa*, *Angpt2,* and *Hif1α* mRNA expression in the retinas was determined by qPCR analysis, *n* = 5. ***p < 0.001 vs. C57BLKS/J mice and ^&^ *p <* 0.05 vs. *db/db* mice by one-way ANOVA with Bonferroni correction.

*db/db* mice are a well-established diabetic mouse model for the study of DR (Hammer et al., 2021). To further detect whether dioscin could inhibit vascular damage *in vivo*, dioscin was administered to mice (oral gavage or ocular delivery; from 8 to 24 weeks of age) every day. To determine this, we initially isolated the retinal network of vessels and conducted PAS staining to assess the effect of dioscin on the retinal vasculature (Figure 5A). In addition, we determined the retinal thickness and structural alterations by H and E staining (Figure 5A). Compared to C57BLKS/J mice, PAS staining revealed numerous acellular capillaries formed in the retinas of *db/db* mice (middle panel of Figure 5A; quantitative results are shown in panel Figure 5B, indicated by the black arrows). However, the formation of acellular capillaries was substantially inhibited by dioscin (right panel of Figure 5A; quantitative results are shown in panel Figure 5B). Similar with these results, when compared with C57BLKS/J mice, whole central retinal thickness was significantly reduced in db/db mice and improved after dioscin intervention. The mRNA levels of angiogenesis-related genes (*Vegfa*, *Angpt2*, *Vegfr2*, and *Hif1a*) were also consistent with our findings (Figure 5C). When compared with C57BLKS/J mice, angiogenesis-related genes were significantly increased in *db/db* mice and significantly decreased after dioscin intervention. Consistent with this, these similar changes were observation after oral administration of dioscin (Figures 6A–D). In addition, dioscin treatment did not influence blood glucose levels in *db/db* mice, suggesting that the improvement of dioscin on retinal abnormality was independent of changes in blood glucose (Figure 6C). Thus, these results in Figure 5 indicate that dioscin protects *db/db* mice against diabetes-induced retinal damage.

## Discussion

In the present study, we explored the HERB database for herbal active ingredients that could potentially target VEGFA, while using molecular docking for compound screening, and identified dioscin as most likely to interact with VEGFA. Next, *in vitro*, we found that dioscin could inhibit the activation of the VEGFA–VEGFR2 signaling pathway and cell proliferation in an HG environment. A more important dioscin intervention inhibits vascular damage in the retinas of *db/db* mice. Taken together, our results show that dioscin may be a potential clinical candidate compound for the treatment of DR.

Dioscin, a steroidal saponin isolated from the Dioscoreaceae family, is an edible plant that is a starchy staple in less-developed countries worldwide (Adedayo et al., 2011). In 2015, dioscin-containing DA-9801 completed a Phase II clinical trial in the US for the treatment of diabetic neuropathy, demonstrating an acceptable safety profile (Kang et al., 2017). Dioscin lowers blood uric acid levels and promotes uric acid clearance, thereby improving renal damage caused by hyperuricemia (Zhang et al., 2018). Dioscin has an inhibitory effect on the formation and development of *Candida* albicans biofilms (Liu et al., 2017). Dioscin has also shown *in vitro* antiviral activity against adenovirus, hepatitis B virus, and blistering stomatitis virus (Liu et al., 2013). Dioscin has antitumor activity against lung, esophagus, stomach, colon, glioblastoma, cervical, ovarian, breast, prostate, leukemia, and many other tumors (Xu et al., 2016). *In vivo*, dioscin inhibits the NF-κB signaling pathway and protects against systemic inflammatory response syndrome (Zhao et al., 2018). Dioscin promotes β-cell proliferation *via* the Wnt/β-catenin pathway and attenuates the decrease in viability and apoptosis of β-cells induced by HG treatment (Yu et al., 2018).

In China, three drugs containing dioscin as the main active component are used to treat cardiovascular diseases: Di’ao Xinxuekang capsules, *Dioscorea saponin* tablets, and Dunyeguanxinning tablets. Among these, Di’ao Xinxuekang capsules have been used for the treatment of coronary heart disease for over 30 years (Qu et al., 2018). According to these studies, dioscin has a therapeutic or adjuvant therapeutic effect against heart disease, but no drug containing dioscin or diosgenin as a single component has been developed for clinical use. To our knowledge, this is the first study to report that dioscin single component treatment could inhibit vascular damage in the retinas of *db/db* mice. At the same time, we first identified dioscin as a VEGFA inhibitor, and dioscin inhibited the transduction of the VEGF signaling pathway as well as VEGFA-mediated cell proliferation in HRMECs.

Yu et al. investigated the subchronic toxicity of dioscin in rats; when dioscin was administered at 300 mg/kg body weight for 90 days, the levels of alanine aminotransferase in both male and female rats increased significantly, which indicated an impairment of liver function; a dose-dependent trend in liver injury was also observed in male rats (Xu et al., 2012). In this scenario, the hepatotoxicity of dioscin may be dose-related. However, in the present study, 80 mg/kg body weight dioscin was used to treat *db/db* mice, and the corresponding rat dose was 55 mg/kg body weight (55 = 80 × 0.69). Clearly, 55 mg/kg body weight is much less than 300 mg/kg body weight. This suggests that the dose of 80 mg/kg body weight dioscin [the human dose is 8.8 mg/kg body weight (8.8 = 80/9.1)] may be relatively safe, but more experiments are required to verify this. Importantly, there are no clinical reports on the hepatotoxicity of traditional Chinese medicinal products containing dioscin.

In conclusion, our study indicates that dioscin inhibits the vascular damage in the retina of *db/db* mice and implies an important potential application of dioscin for treatment of DR in clinics.

## Data Availability

The original contributions presented in the study are included in the article/Supplementary Material; further inquiries can be directed to the corresponding author.

## Abbreviations

B7-H3, B7 homolog 3; CIE, -CDOCKER interaction energy; DR, diabetic retinopathy; ECs, endothelial cells; FOXO1, Forkhead box protein O1A; HG, high glucose; VEGF, vascular endothelial growth factor; VEGFR, vascular endothelial growth factor receptor; VEGFR1, vascular endothelial growth factor receptor 1; VEGFR2, vascular endothelial growth factor receptor 2.
