# Supplementary material for: Dioscin Reduces Vascular Damage in the Retina of db/db Mice by Inhibiting the VEGFA Signaling Pathway
Source: Front Pharmacol. 2022 Jan 28;12:811897. doi: 10.3389/fphar.2021.811897 (PMC8832152; doi:10.3389/fphar.2021.811897)

Dioscin inhibit angiogenesis in *db/db* mice by inhibiting VEGFA signaling pathway

Jun Wang[1], Guang Yan Yang[1], Hong Yan Sun[2], Ting Meng[2], Chu Chu Cheng[1], Hui Pan Zhao[2], Xiao Ling Luo[2], Ming Ming Yang[2,*]

Supplementary figures (the whole uncropped images of the original western blots)

Figure 1C VEGFA

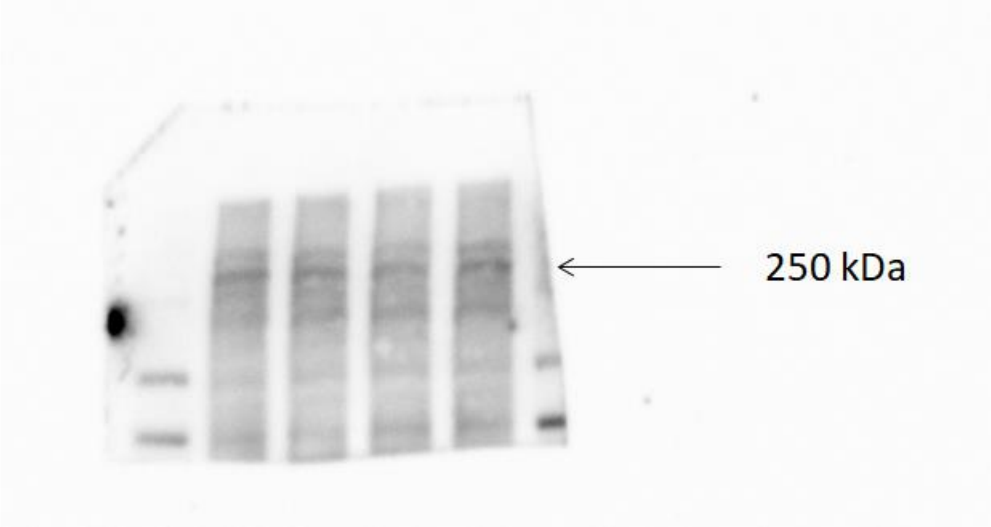

Figure 1C p-VEGFR2

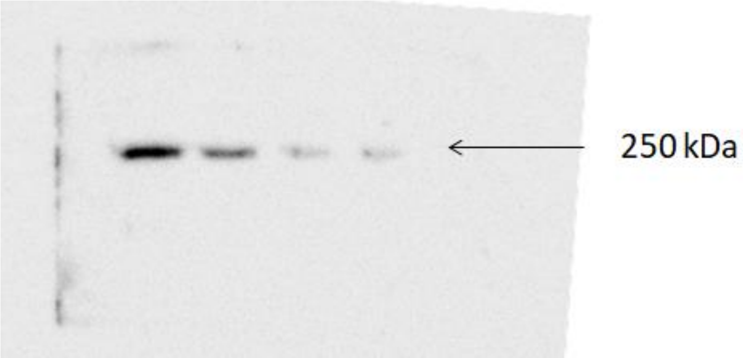

Figure 1C VEGFR2

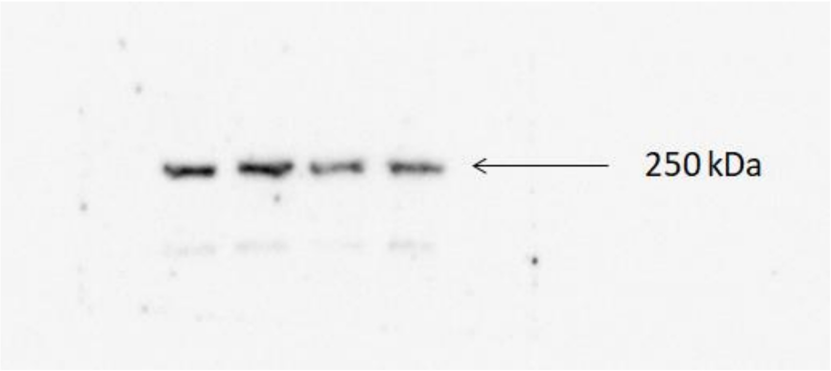

Figure 1C β-actin

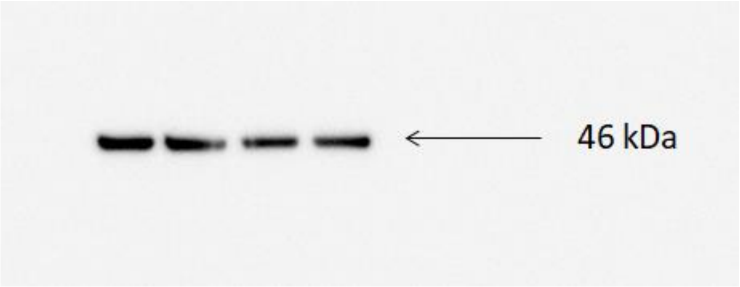

Figure 2A p-ERK1/2

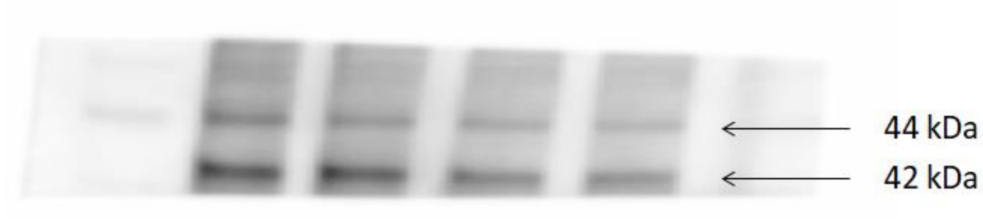

Figure 2A ERK1/2

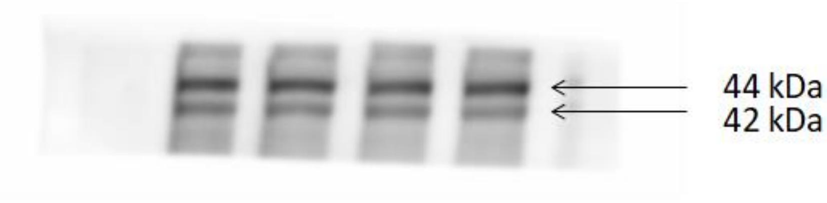

Figure 2A p-Akt

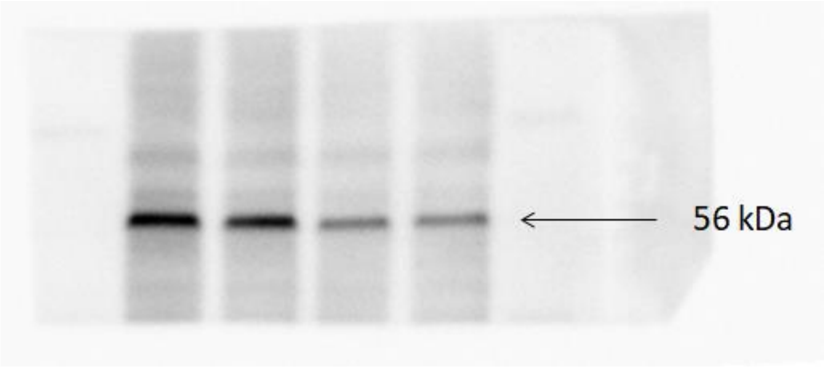

Figure 2A Akt

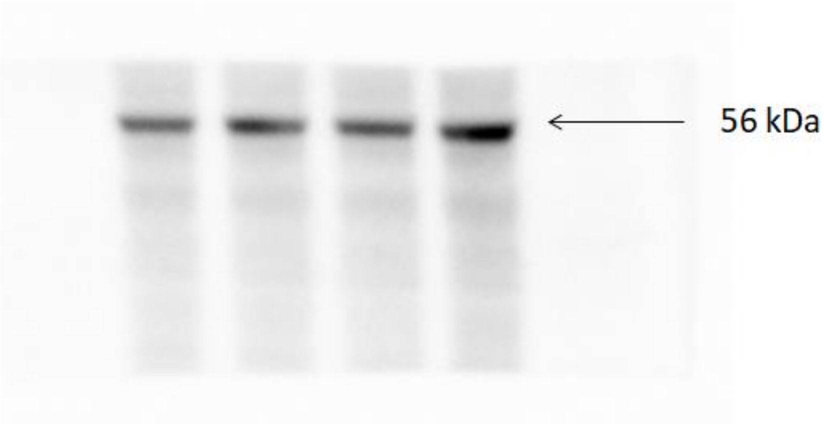

Figure 2A β-actin

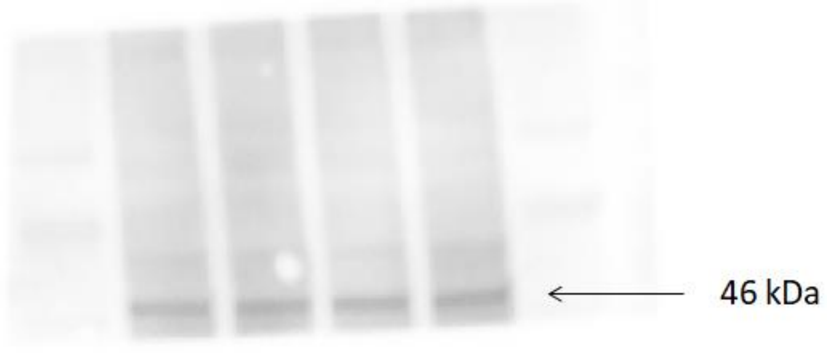

Figure 3 VEGFR2

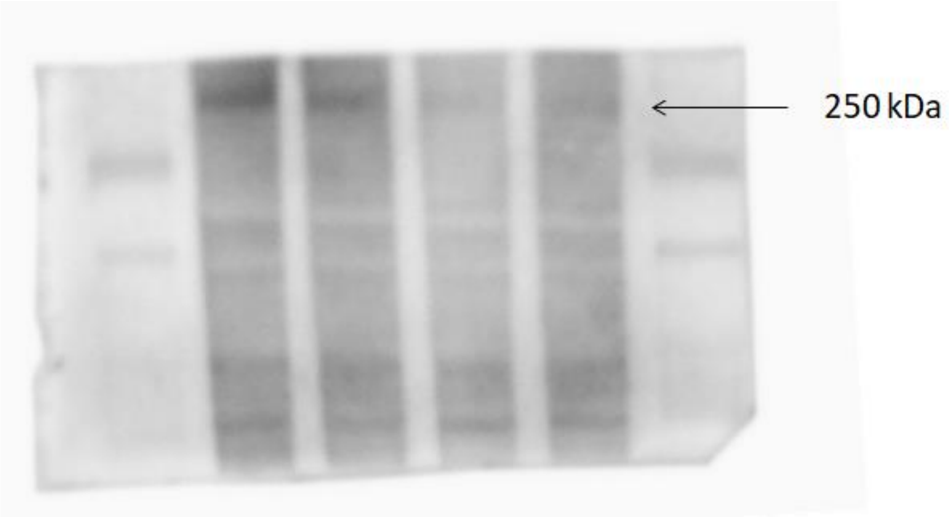

Figure 3 VEGFA

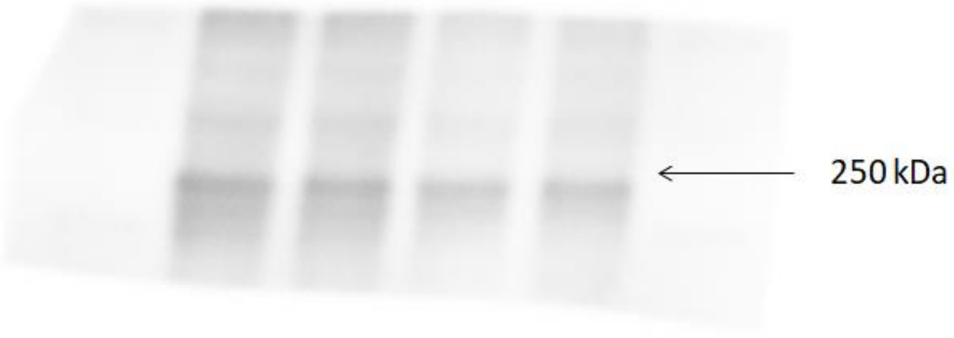

Figure 4A VEGFA

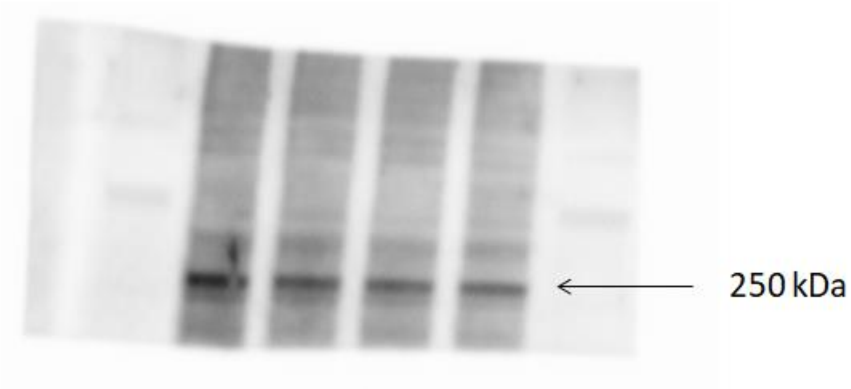

Figure 4A p-VEGFR2

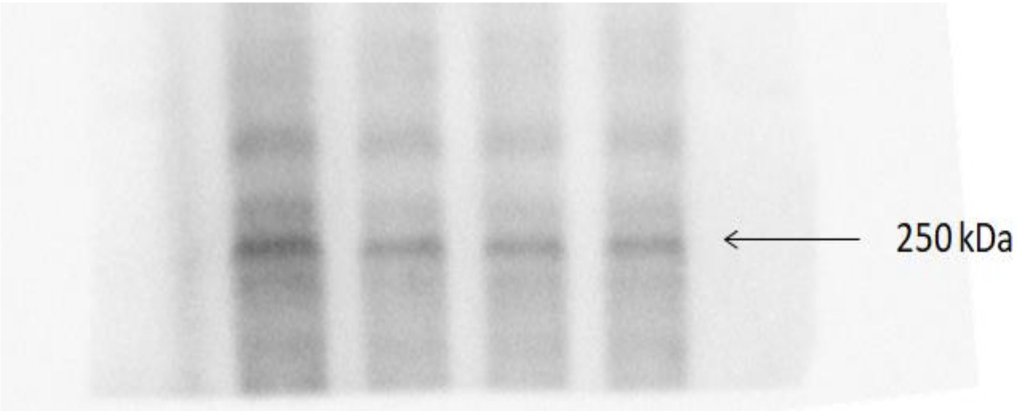

Figure 4A VEGFR2

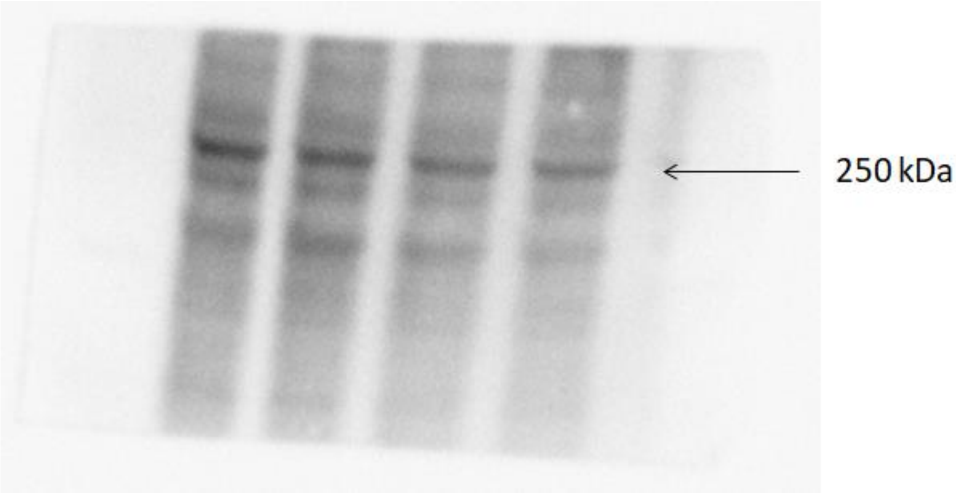

Figure 4A p-Akt

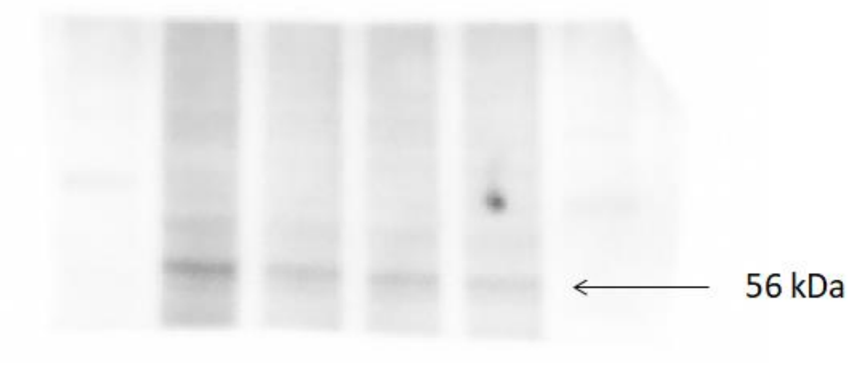

Figure 4A Akt

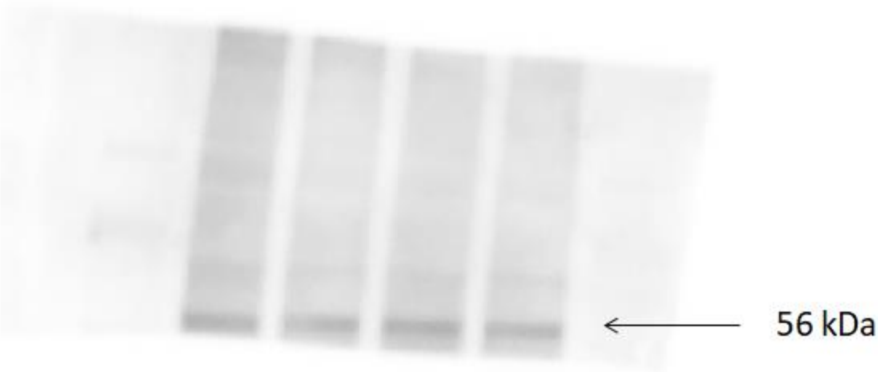

Figure 4A b-actin

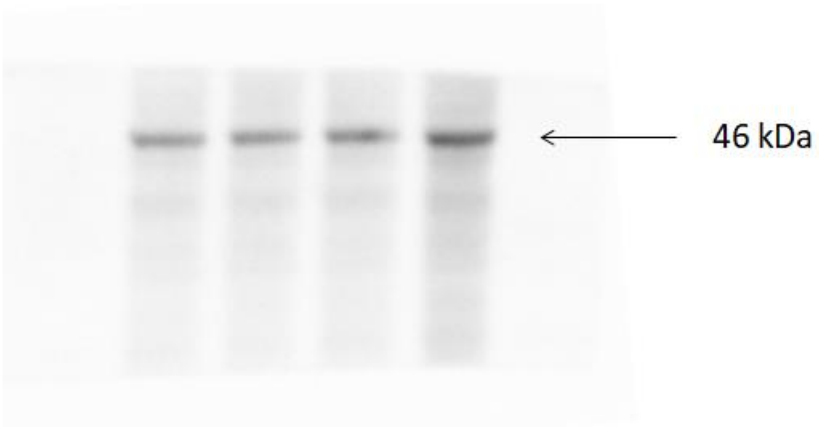

Supplement: Supplementary file 1 [file DataSheet1.PDF]
